# *Add Sugar to Chitosan:* Mucoadhesion and In Vitro Intestinal Permeability of Mannosylated Chitosan Nanocarriers

**DOI:** 10.3390/pharmaceutics14040830

**Published:** 2022-04-11

**Authors:** Sadaf Ejaz, Bridget Hogg, Delyan R. Hristov, David J. Brayden, Muhammad Imran, Sourav Bhattacharjee

**Affiliations:** 1 Department of Biosciences, COMSATS University Islamabad (CUI), Park Road, Islamabad 45550, Pakistan; sadaf_ejaz@hotmail.com; 2 School of Veterinary Medicine, University College Dublin (UCD), Belfield, D04 W6F6 Dublin, Ireland; bridget.hogg@ucd.ie (B.H.); delyan.hristov1@ucd.ie (D.R.H.); david.brayden@ucd.ie (D.J.B.); 3 Conway Institute of Biomolecular and Biomedical Research, University College Dublin (UCD), Belfield, D04 V1W8 Dublin, Ireland

**Keywords:** chitosan nanoparticles, mannosylation, surface functionalization, mucoadhesion, surface charge density, bioconjugation

## Abstract

Crosslinked chitosan nanocarriers (140–160 nm) entrapping coumarin-6 (*λ_ex/em_* = 455/508 nm) with or without surface mannosylation were synthesized and assessed for cytotoxicity, adherence and cellular uptake in Caco-2 cells, flux across Caco-2 monolayers, and mucoadhesion to porcine mucin. Mannosylated and non-mannosylated nanocarriers demonstrated biocompatibility with slow release of coumarin-6 at pH 6.8 and 7.4 over 24 h. Adherence of the non-mannosylated nanocarriers (50 and 150 µg/mL) to Caco-2 cells was ~10% over 24 h, whereas cellular uptake of 25–30% was noted at 4 h. The mannosylated nanocarriers showed a similar adherence to non-mannosylated nanocarriers after 24 h, but a lower cellular uptake (~20%) at 1 h, comparable uptake at 4 h, and a higher uptake (~25–30%) at 24 h. Overall, the nanocarriers did not affect the integrity of Caco-2 monolayers. Mannosylated nanocarriers elicited higher P_app_ of 1.6 × 10^−6^ cm/s (50 µg/mL) and 1.2 × 10^−6^ (150 µg/mL) than the non-mannosylated ones: 9.8 × 10^−7^ cm/s (50 µg/mL) and 1.0 × 10^−6^ (150 µg/mL) after 2 h. Non-mannosylated chitosan nanocarriers elicited enhanced adhesion to porcine gut mucin *via* mucin-filled microchannels due to higher cationic charge density. These results underpin the importance of surface chemistry in the biological interactions of nanocarriers, while highlighting the role of surface hydrophilicity in mucopermeation due to mannosylation.

## 1. Introduction

Low bioavailability of orally administered drugs has long impeded translation, but nano-drug delivery systems (nano-DDSs) can potentially be used to improve bioavailability [1]. Downsizing the particulate carriers from micro- to nanoscale may improve the potential further; however, drug absorption with dosing precision across intestinal regions remains a challenge. Great advances in material science, particularly in polymer engineering, have assisted in designing advanced nanocarriers (NCs) that can exploit localized anatomic and physiologic attributes of the human intestine to facilitate controlled release of an entrapped payload [2,3].

The mucus layer above the small intestinal epithelial brush border presents a challenge for oral drug delivery [4]. The protective mucus layer is also present in different compositions in the eyes, reproductive system, and respiratory tracts [5]. The intestinal mucus hydrogel is mainly composed of mucin biopolymer, contributing to ~90% of its solid components [6]. The remaining solid materials in the mucus matrix are cells, debris, and genetic materials. Additionally, the microbiomes form a significant part of the intestinal tract [7]. Rich in glycoproteins, mucus can absorb water in order to swell, while its viscoelasticity varies based on the pathophysiologic state, age, food intake, and infections. The human gut mucus layer is recycled every 4–6 h in the body [8].

Gut mucus presents a formidable barrier against the transport of NCs trying to reach the epithelial layer to either release the encapsulated drug or be endocytosed by epithelia. It has evolved into a highly selective sieve that is difficult to permeate or mimic, either In Vitro or ex vivo. Gut mucus intercepts NCs as foreign entities and rapidly filters them out by first adhering to the NCs and removing them with recycled mucus. Such mucoadhesion is driven by polyvalent interactions, hydrophobic bonding, formation of hydrogen bonds, Van der Waal forces, and π–π stacking [9,10].

Ideally, NCs made up of mucoadhesive materials might adhere to the gut epithelial layer, where systemic drug absorption is initiated. The mechanism of NC uptake, with the encapsulated cargo of drug molecules, nevertheless remains unclear. It probably occurs as a combination of multiple processes, from the uptake of intact NCs to a complete dissolution at the vicinity of the gut wall followed by an uptake of free drug. In both cases, a mucoadhesive drug-entrapped nanoformulation helps in facilitating the transport of drug molecules across the gut epithelia [11].

Chitosan is a cationic, natural, and biocompatible biopolymer [12], with its chemistry well worked out, particularly from an encapsulation and bioconjugation perspective. The cationic charge of chitosan facilitates its binding to the negative sites in mucin. Modification of chitosan at its primary or secondary hydroxyl and amine/acetamide groups can be achieved without harsh synthetic conditions [13]. However, such modifications alter the surface properties of chitosan NCs (CNCs) and influence their physicochemical properties. It is established now that the surface properties, such as hydrophilicity and surface charge, determine the behavior of NCs within a biological milieu [14,15].

CNCs have emerged as a nano-DDS with delivery potential in the GI tract [16]. Furthermore, surface-functionalized CNCs exhibit tunable physicochemical properties. For example, *p*-coumaric-acid-conjugated CNCs elicited 1.6-fold higher mucoadhesion when compared to native CNCs [17]. On the other hand, the surface chemistry of chitosan can be exploited to develop *stealth* NCs that can efficiently permeate a layer of mucus [18]. Such stealth CNCs are typically functionalized with hydrophilic molecules, such as polyethylene glycol [19] or cyclodextrin [20]. Similarly, quaternization of the primary amine group in chitosan and synthesis of thiolated chitosan, carboxymethyl chitosan, and *N*-succinyl chitosan have been explored as oral nano-DDSs with beneficial features, including increased mucoadhesion and decreased sensitivity to metabolic enzymes in gut [21].

Mannose, a C2 epimer of glucose, is an important sugar that participates in metabolism, including glycosylation [22]. Its hydroxyl groups impart hydrophilicity to the molecule and may render stealth properties when grafted onto NCs—an observation that holds promise toward developing mannose-grafted CNCs (M-CNCs) with augmented mucopermeability. Surface grafting of mannose also enables targeting of mannose receptors expressed on the dendritic cells and macrophages [23]. Moreover, its affordability and biocompatibility make it a good choice for bioconjugation.

Synthesis of M-CNCs has been reported [24], albeit systematic studies on how it behaves within biological interfaces, especially from a drug delivery perspective, remain sparse. To this end, CNCs and M-CNCs with an entrapped fluorophore, coumarin-6 (C6), (C6@CNC and C6@M-CNC) were prepared and investigated for release studies, cytotoxicity, and mucopermeation in terms of flow through mucin-filled microchannels. The data provide a facile synthetic route for preparing M-CNCs, with further insights into how the grafting of hydrophilic sugar molecules on CNCs affects mucopermeation. Finally, we identified key steps to help prepare functionalized CNCs with encapsulated biomacromolecules, which may facilitate drug delivery.

## 2. Materials and Methods

### 2.1. Chemicals and Reagents

Medium molecular weight chitosan (88.3% deacetylated; CAS number: 9012-76-4; Lot No.: 281219) was obtained from ChitOcean Inc. (St John’s, NL, Canada). Fetal bovine serum (FBS), phosphate-buffered saline (PBS), non-essential amino acid (NEAA, product code 11140035), Hank’s balanced salt solution (HBSS, H8264), 2-(*N*-morpholino)ethanesulfonic acid (MES) buffer, 0.25% (*w*/*v*) trypsin, ethylenediaminetetraacetic acid (EDTA) solution (0.02% in 0.5 mM DPBS), Dulbecco’s modified eagle medium (DMEM, product code D6546), penicillin-streptomycin (product code P4333), L-glutamine, D-(+)-mannose (product code M8574), sodium pentatripolyphosphate (TPP, product code 72061), sodium triacetoxyborohydride (product code 316393), coumarin-6 (C6, product code 442631), and Triton^®^-X-100 were purchased from Sigma Aldrich (Arklow, Ireland).

### 2.2. Caco-2 Cells

Human colonic adenocarcinoma-derived Caco-2 cells were obtained from the American Type Culture Collection. The cells were grown in 75 cm^2^ flasks with DMEM (supplemented with 10% FBS, 1% NEAA, and 1% L-glutamine) under 5% CO_2_ (37 °C) and passaged at 70–80% confluency. Before seeding, cell counting (with trypan blue staining as a viability check; cells had viability of >90%) was performed using Countess^®^ automated cell counter. Passaging was carried out using 0.05% trypsin-EDTA followed by centrifugation at 300× *g* and resuspension in complete media before counting.

### 2.3. Synthesis of C6@CNCs and C6@M-CNCs

**Figure 1:**
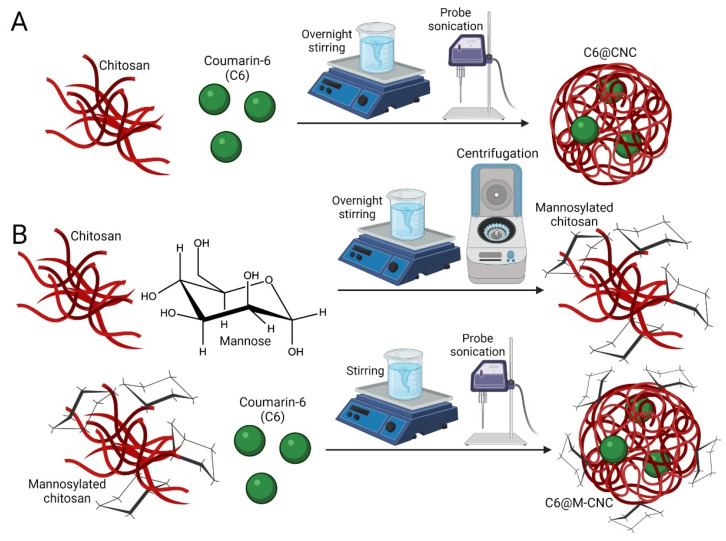
Reaction scheme for preparing: (**A**) C6@CNCs and (**B**) C6@M-CNCs.

Chitosan solution (4.7 mg/mL) in 2% acetic acid was left stirring overnight in a flask (Figure 1A). Solutions of mannose (0.027 g/10 mL) and sodium triacetoxyborohydride (0.1 M) were prepared and stirred at 100 rpm for 30–40 min before being added 1:1 (*v*/*v*) to the chitosan solution (pH 5–6). Solutions were left stirring for 48–72 h (100–200 rpm on a plate stirrer) for conjugation (Figure 1B). The mannosylated chitosan solution was subjected to 1N NaOH treatment followed by centrifugation at 18,000× *g* for 15–20 min. The pellet was resuspended in an equal volume of 0.2–0.5% acetic acid and stirred till complete dissolution. Subsequently, fresh 0.5% TPP solution was added dropwise (1–2 mL/10 mL), and the mixture was stirred for another 30 min to prepare a crosslinked matrix. Only C6 (1 mg/mL in 1:9 chloroform:ethanol) was added before TPP and stirred for 20 min at 100 rpm on a plate stirrer for fluorophore encapsulation. The colloidal dispersion was sonicated for 30–40 min in an ultraprobe sonicator followed by freeze-drying and analyses. CNCs and M-CNCs without encapsulated C6 were prepared as controls.

The loading efficiency (LE) was calculated as:

$$\mathrm{LE}\left(\%\right)=\frac{\mathrm{Total}\;\mathrm{encapsulated}\;C6\;\mathrm{payload}}{\mathrm{Total}\;\mathrm{chitosan}\;\left(\mathrm{unconjugated}\;\mathrm{or}\;\mathrm{mannosylated}\right)}\times 100$$

### 2.4. Nuclear Magnetic Resonance (NMR) Analyses

Chitosan, with or without mannosylation, was characterized with ^1^H NMR. Chitosan samples were dissolved in D_2_O, while mannosylated chitosan was dissolved in 1% d_4_-acetic acid (CD_3_COOD) in D_2_O. Mannose was dissolved in D_2_O. The NMR spectra were recorded in a Varian VnmrS spectrometer (600 MHz) at 25 °C. Receiver gain was set to automatic and was between 32 and 42. The relaxation delay was 2 s while the acquisition time was 5 s. The spectra were analyzed using MastreNova 14.2.0 software (Mastrelab Research). All peaks were referenced to the residual solvent peak. A first-order Bernstein polynomial fit was used as the baselining method. No apodization or zero filling was utilized.

### 2.5. Scanning Electron Microscopy of C6@CNCs and C6@M-CNCs

Drops of C6@CNC and C6@M-CNC dispersions (1 mg/mL) were deposited on an aluminum foil and left for overnight drying. The foil with deposited NCs was then fixed on a stub, followed by sputter-coating with gold. The imaging was performed using a Hitachi S-4300 scanning electron microscope.

### 2.6. Hydrodynamic Size Determination of C6@CNCs and C6@M-CNCs

Hydrodynamic diameters of the NC dispersions (100 µg/mL) were measured by dynamic light scattering (DLS) using a Malvern Zetasizer Nano ZS (*λ_ex_* = 633 nm) instrument. Two independent measurements were performed in a clear disposable sizing cuvette at 25 °C and 173° backscatter angles for each sample.

### 2.7. C6 Quantification

The quantification of C6 was performed using a microplate reader (Spectra max Gemini, Molecular Devices, *λ_ex/em_* = 455/508 nm) using serial dilutions (0.78 μg/mL–0.76 ng/mL) of C6 in a 9:1 mixture of ethanol/chloroform (Supplementary Material S1).

### 2.8. Release of C6 from C6@CNCs and C6@M-CNCs

In Vitro C6 release studies from C6@CNCs and C6@M-CNCs at 0.78 µg/mL concentration in HBSS-HEPES (pH 7.5) and HBSS-MES (pH 6.8) were conducted. The NCs were suspended in the buffers (1:1) at 37 °C and under constant stirring of 100 rpm. Aliquots were collected at predefined intervals (*t* = 0, 0.5, 1, 1.5, 2, 2.5, 3, 3.5, 4, 4.5, 5, and 24 h) and subjected to centrifugation (18,000× *g* for 15 min, 4 °C). The free C6 content in supernatant was quantified by spectrofluorometry (*λ_ex/em_* = 455/508 nm), while cumulative release was estimated after 5–24 h.

### 2.9. MTS Assay

The Caco-2 cells were seeded in a 96-well plate (0.5 × 10^5^ cells/mL, 200 µL/per well) and incubated for 24 h at 37 °C and 5% CO_2_. The medium was discarded next day, and the cells were washed twice with PBS followed by equilibration for 30 min with HBSS (2.5 M glucose and buffered with 25 mM MES). Afterward, the cells were treated with the NCs in HBSS/MES for 4 h. Later, the fluid in each well was aspirated, followed by washing (2×) with PBS. The MTS reagent (CellTiter 96^®^ AQueous One–Promega (Madison, WI, USA); product number G3582) was then added to the wells according to the manufacturer’s instructions, and the cells were incubated for a further 4 h. Cells in media without NCs, cells in media, and cells exposed to Triton-X (0.001%) were used as controls. The plate was then read in a microplate reader (UVM 340 plate reader, ASYS Hitech Gmbh, Austria) at 490 nm wavelength. The data were expressed as a % of the negative control.

### 2.10. Caco-2 Adherence and Uptake of CNCs

The Caco-2 cells were seeded on six-well plates (1 × 10^5^ cells/mL, 2 mL per well, passage 26–36) after coating with type II rat tail collagen to enhance cell adhesion. Upon seeding, the culture media were replaced every other day for 21 days, and the plate was incubated (37 °C, 5% CO_2_) to allow the cells to form monolayers. Dispersions of C6@M-CNCs and C6@CNCs with a loading of ~0.64 ng C6/µg NCs (50 µg/mL and 150 µg/mL) and free C6 suspensions were prepared in HBSS (2.5 M glucose and buffered with 25 mM MES). The monolayers were rinsed and equilibrated with HBSS for 30–60 min at 37 °C and 5% CO_2_ before the exposure. The buffer was later replaced with test samples in HBSS, and the monolayers were incubated for different time intervals (*t* = 1, 4, and 24 h) at 37 °C in a shaking incubator. The supernatant was collected at each time point and analyzed with spectrofluorometry (*λ_ex/em_* = 455/508 nm). Furthermore, the cells were treated with 0.2% Triton-X 100 to cause cell lysis for 2 h, and cell debris was removed by centrifugation (18,000× *g* for 15 min) followed by the spectrofluorometric analysis. The obtained data were used to calculate % cellular uptake, while the adherence (µg/cm^2^) was calculated from the difference of spectrofluorometric reading of total NCs, and the sum of free and internalized NCs. Experimental controls were as follows: free C6 (same concentration as in NCs), HBSS (buffered), cells with and without test sample. The fluorescence intensity of the wells with cells alone was used as a negative control, while those with the test sample were used as a positive control. The following equation was used to measure cellular uptake:

$$\%\;\mathrm{Uptake}\;\mathrm{efficiency}=\frac{\mathrm{test}\;\mathrm{sample}-\mathrm{negative}\;\mathrm{control}}{\mathrm{positive}\;\mathrm{control}-\mathrm{negative}\;\mathrm{control}}\times 100\%$$

### 2.11. Transepithelial Flux of C6 in NCs across Caco-2 Monolayers

Transport of the test samples (C6@M-CNCs and C6@CNCs) and free C6 was measured across filter-grown Caco-2 monolayers (Supplementary Material S2). Cells were seeded at a density of 0.6 × 10^6^ cells/mL; 500 µL per well on Transwell^®^ polycarbonate filters (Costar-Corning Ltd., pore size 0.4 µm, surface area for cellular growth 1.12 cm^2^) with 0.5 and 1.5 mL medium in apical and basolateral compartments, respectively, followed by incubation (37 °C, 5% CO_2_) for 21 days. The viability of the cells was checked by epifluorescence after staining the nuclei with Hoechst stain (*λ_ex/em_ =* 470/525 nm). The media were replaced on the apical side 24 h after seeding, and then both the apical and basal media were replaced every two days. The integrity of the cell monolayers on filters was determined by transepithelial electrical resistance (TEER), and transport experiments were conducted on Caco-2 monolayers with TEER ≥ 2000 Ω·cm^2^. Before the experiment, the cell culture medium (apical and basolateral) was replaced with HBSS and incubated at 37 °C under 5% CO_2_ for 30 min. The buffer in the apical side of the filters was then replaced with HBSS containing test samples while permeability studies were conducted at 37 °C and 5% CO_2_ for 2 h, with samples collected from the basolateral side at *t* = 0, 0.5, 1, 1.5, and 2 h. The TEER was monitored 30 min before and following equilibrium in HBSS before exposure to ensure the stability of the TEER, and later recorded at *t* = 0, 0.5, 1, 1.5, 2, 4, and 24 h. The apparent permeability coefficient (P_app_) of C6 was calculated by the following equation:

$$P_{\mathrm{app}}=\frac{\mathrm{dQ}}{\mathrm{dt}}\times \frac{1}{A\times C_{0}}$$

where dQ/dt = flux at time *t*, A = surface area of the transwell membrane, and C_0_ = initial concentration of C6 in the apical compartment.

### 2.12. Purification of Porcine Gut Mucin

Mucin was harvested from abattoir-sourced samples of porcine gut within 6 h of dispatch and was mixed with an equal volume of 8M guanidine hydrochloride (Sigma Aldrich) to solubilize and homogenize the mucus samples. Dithiothreitol (Sigma Aldrich) was added to a final concentration of 10 mM and incubated at 37 °C for 5 h. Sodium iodoacetamide (Sigma Aldrich) was added to a final concentration of 25 mM, and the samples were incubated at room temperature overnight. Isopycnic density gradient centrifugation was carried out by adjusting the density of the sample to 1.40 g/mL with solid cesium chloride, and then placing the sample in Beckman Ultra-clear tubes which were centrifuged at 65,000 rpm × 18 h at 10 °C in an Optima L-100 XP (Beckman Coulter Inc., Brea, CA, USA) ultracentrifuge using the 70 Ti rotor without any break. The density gradient created was unpacked in 1 mL fractions from the top to the bottom of the tube. 

Duplicate 5 μL aliquots from each fraction were then blotted onto a polyvinylidene difluoride membrane using a Whatman manifold Slot blot apparatus (Schleicher & Schuell, Inc, Keene, NH) and stained with periodic acid-Schiff stain (PAS, VWR, Radnor, PA, USA) to assess the relative intensity of carbohydrate. The carbohydrate-rich fractions observed from the slot blot, which had a density profile of between 1.35 to 1.45 g/mL, were pooled and separated by size exclusion chromatography. The samples were loaded on a Sepharose CL-4B column (Sigma Aldrich) and eluted with 50 mM Tris/100 mM KCl, pH 7.5, as mobile phase. The elute was collected in fractions of 4 mL using a fraction collector, and then a sample of all fractions was slot blotted and stained with the PAS stain. Mucin-rich fractions were pooled and freeze-dried, then resuspended in water and desalted on a Sephadex G-25 column (Sigma Aldrich). Fractions collected with the fraction collector were analyzed by PAS staining (~40 mL of sample), then carbohydrate-rich fractions were pooled and freeze-dried before being stored (1 mg/mL) at −20 °C in sterile water.

### 2.13. Mucoadhesion Studies

**Figure 2:**
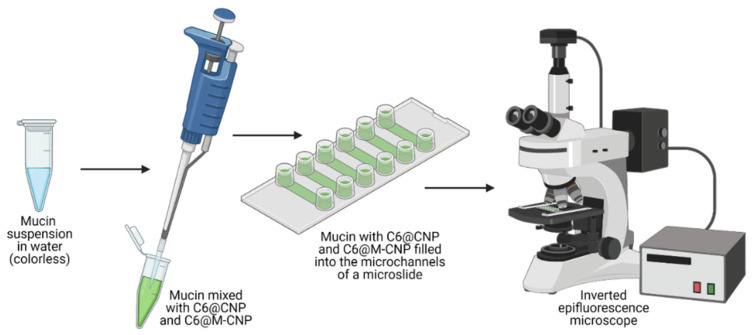
The protocol of mucoadhesion studies. Purified porcine gut mucin was dispersed in water (1 mg/mL), aliquoted to an Eppendorf tube, and mixed with C6@CNC or C6@M-CNC suspension (150 µg/mL). The mixture was then injected into the microchannels of an optically translucent microslide with six parallel microchannels before being imaged in an inverted microscope (*λ_ex/em_* = 470/525 nm).

An optically translucent microslide (µ-Slide VI 0.1; Ibidi GmbH; Figure 2) containing six parallel microfluidic channels (*l × b × h* = 17 × 1 × 0.1 mm^3^; volume 1.7 µL) with reservoirs (60 µL) attached at both ends of each microchannel was used. A 90 µL aliquot of mucin in water (1 mg/mL) was mixed with 10 µL of C6@M-CNCs or C6@CNCs in buffer (150 µg/mL), and 30 µL of the mixture was deposited into an end-reservoir of the microchannels. Gentle suction pressure with the help of a syringe was applied to the opposite end-reservoir to fill the microchannels with the mucin-NC mixture. The filled-out microchannels were then imaged (*λ_ex/em_* = 470/525 nm) in an inverted Zeiss Axiovert 200 M epifluorescence microscope (NA0.25 lens and Zeiss filter set 43) at 37 °C and for 100 cycles of 5.5 s exposure at 10 s intervals (*n* = 3).

### 2.14. Statistical Analyses

All experiments were done in triplicates (*n* = 3) and data presented as mean ± standard error of the mean (SEM). Data points were expressed as a % of negative control, and unpaired Student’s *t*-tests were performed. Data points were considered to be significantly different at the *p* < 0.05 level.

## 3. Results and Discussion

### 3.1. Mannosylation of Chitosan

**Figure 3:**
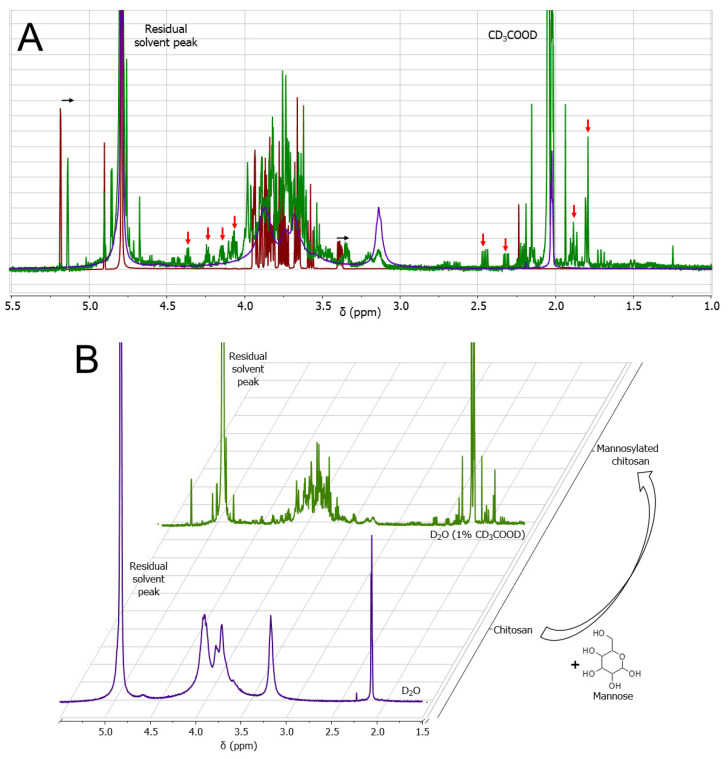
(**A**) Overlapped ^1^H NMR spectra of chitosan (purple), mannose (red), and mannosylated chitosan (green) with highlighted changes due to mannosylation. Red and black arrows indicate new peaks and peak shifts, respectively. (**B**) Stacked chitosan and mannosylated chitosan ^1^H NMR spectra showing the difference in peak broadening and presence of fine structure post-mannosylation.

The mannosylation reaction was sensitive to subtle variations of pH, and firm control over the pH of the reaction mixture was critical. Hence, a continuous monitoring of pH was necessary. Furthermore, the overnight stirring of chitosan solution in 2% acetic acid before the reaction to achieve complete dissolution is an important step toward adequate mannosylation. The mannose, chitosan, and mannosylated chitosan were analyzed by ^1^H NMR (Figure 3). Chitosan and mannose peaks were consistent with literature data (Supplementary Material S3) [25,26,27]. Two major changes were observed in mannosylated chitosan compared to the unconjugated chitosan. Firstly, the mannose peaks shifted upfield by 0.045 ppm (Figure 3A). This is potentially due to the addition of CD_3_COOD. Secondly, upon mannosylation, the fine structure of chitosan became visible in the spectra, including the appearance of new peaks (Figure 3B). Signal broadening, such as that of chitosan in D_2_O (bottom spectra, Figure 3B), is complex and may be caused by a variety of reasons [28]. In this case, a likely contribution is the low chitosan solubility in water causing it to form a dense Gaussian coil and, thus, to relax more rapidly in NMR. In this context, the appearance of finer structures may indicate a rather favorable D_2_O-chitosan interaction leading to a more open coil. The fine structure is not apparent in chitosan solutions in CD_3_COOD; however, it has been observed with an addition of other sugars to the chitosan backbone [29].

### 3.2. Size of C6@CNCs and C6@M-CNCs

**Figure 4:**
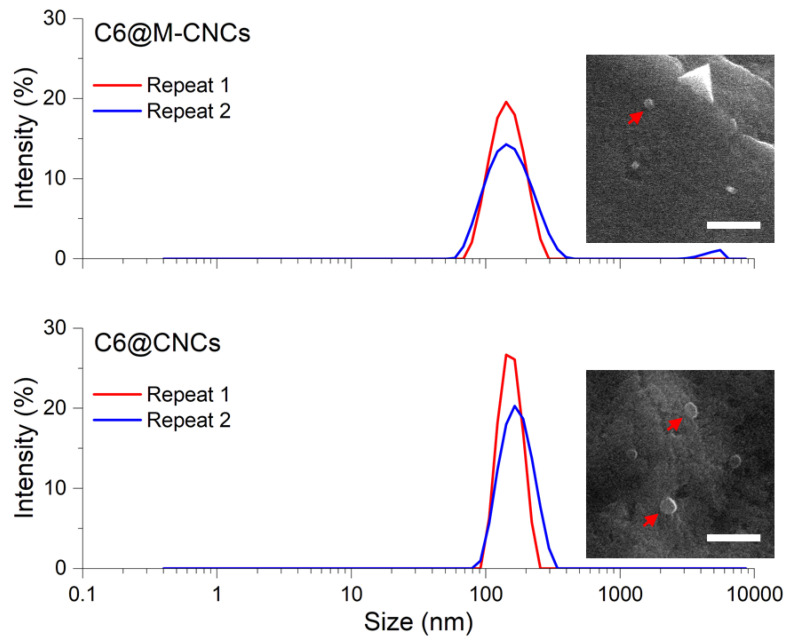
Size determination of the C6@CNCs and C6@M-CNCs was performed with DLS and scanning electron microscopy. The mean hydrodynamic diameters of the C6@CNCs and C6@M-CNCs, as determined by DLS, were 160 nm and 140 nm, respectively. Results from two independent repeats are shown. The scanning electron microscopy images are provided as insets where red arrows mark individual particles. Scale bars denote 1 µm.

The sizes of C6@CNCs and C6@M-CNCs were determined by DLS and scanning electron microscopy. The C6@CNCs were spherical and had a hydrodynamic diameter of 160 ± 15 nm (Figure 4). The C6@M-CNCs were slightly smaller (140 ± 15 nm) which was due to an increased hydrophilicity imparted by mannosylation [30]. The aqueous dispersions of the NCs were stable and did not show any visible agglomeration (25 °C) for at least a week.

### 3.3. Loading Efficiency (LE) of C6 in C6@CNCs and C6@M-CNCs

The C6 was quantified (at 0.78 µg/mL–0.76 ng/mL) in a microplate reader (*λ_ex/em_* = 455/508 nm) using the calibration curve (Supplementary Material S1). The LE was ~99% for C6@CNCs and C6@M-CNCs, while the entrapment efficiencies were 97.2 ± 7.4% and 72.4 ± 5.8%, respectively (Supplementary Material S4). CNCs are known to elicit efficient encapsulation of both hydrophilic [31] and hydrophobic [32] drug payloads due to matrix formation. In this study, the high LE of both the C6@CNCs and C6@M-CNCs was due to a crosslinked matrix and the low concentration of C6 used. Intriguingly, mannosylation did not cause any significant impact on entrapment efficiency, although a difference was observed in the LE. As a hydrophobic molecule, C6 interacts with the polymeric phase at the acetyl group of chitosan and becomes entangled in the polysaccharide chain. Mannosylation also increased steric hindrance and decreased LE for the C6@M-CNCs.

### 3.4. Release of C6 from C6@CNCs and C6@M-CNCs

**Figure 5:**
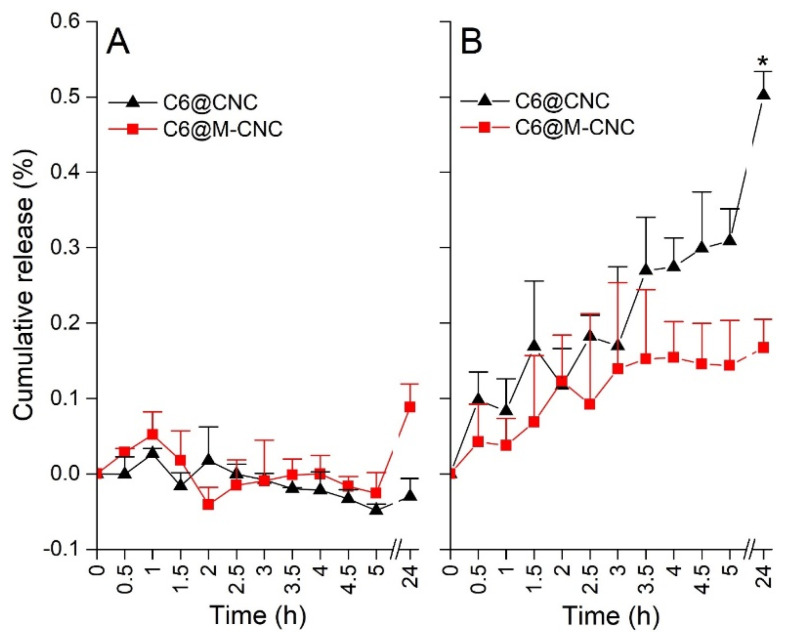
The release kinetics of encapsulated C6 from C6@CNCs and C6@M-CNCs in (**A**) HBSS-HEPES (pH 7.5) and (**B**) HBSS-MES (pH 6.8) buffers. Results are shown as mean ± SEM (*n* = 3). Both the C6@CNCs and C6@M-CNCs showed release in HBSS-MES, with C6@CNCs demonstrating significantly (*p* < 0.05) more release than C6@M-CNCs at 24 h (data point marked with an asterisk).

In Vitro C6 release studies demonstrated <0.3% release from both the NCs at 4 h in HBSS-HEPES and HBSS-MES buffers (Figure 5), while a significant difference was noted after 24 h (C6@M-CNC > C6@CNCs). Moreover, greater release for both the NC prototypes was noted in HBSS-MES, suggesting higher release at acidic pH. Interestingly, the pH 6.8 of the HBSS-MES buffer aligns with that of the human jejunal contents, demonstrating the potential of these NCs as an agent for jejunum-targeted drug release. Burst release of encapsulated molecules from CNCs has been reported before [33]. To restrict such bursts, crosslinked CNCs were made for this study. The pH influenced drug diffusion and swelling is a process that is influenced by amine groups in the chitosan polysaccharide chain. On the contrary, in mannosylated chitosan, at acidic pH values of MES, the number of free amine groups was reduced, thereby alleviating polar interactions.

### 3.5. MTS Assay of CNCs

**Figure 6:**
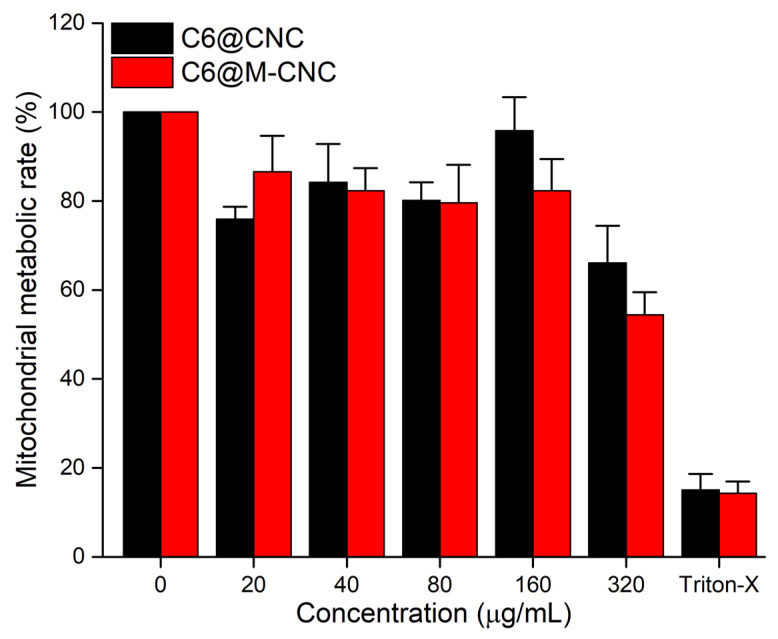
MTS assay of C6@CNCs and C6@M-CNCs (0–320 µg/mL) in Caco-2 cells. Triton^®^-X (0.001%) was the positive control. Results are expressed as a % of negative control (0 µg/mL) and plotted as mean ± SEM (*n* = 3).

Caco-2 cells were exposed to 20–320 µg/mL of C6@CNCs and C6@M-CNCs for 4 h followed by assessment of mitochondrial metabolic activity with the MTS assay (Figure 6). A concentration-dependent decrease in cellular mitochondrial metabolism was observed for both C6@CNC and C6@M-CNC. Moreover, 20–160 µg/mL dilutions of C6@M-CNCs demonstrated ~80% cell viability, while a decreased viability (~60% of negative control) was noted at 320 µg/mL which might have been caused by NC aggregation [34]. Overall, effects were subtle at concentrations below 160 µg/mL.

### 3.6. Adherence and Uptake Analyses of C6@CNCs and C6@M-CNCs in Caco-2 Cells

**Figure 7:**
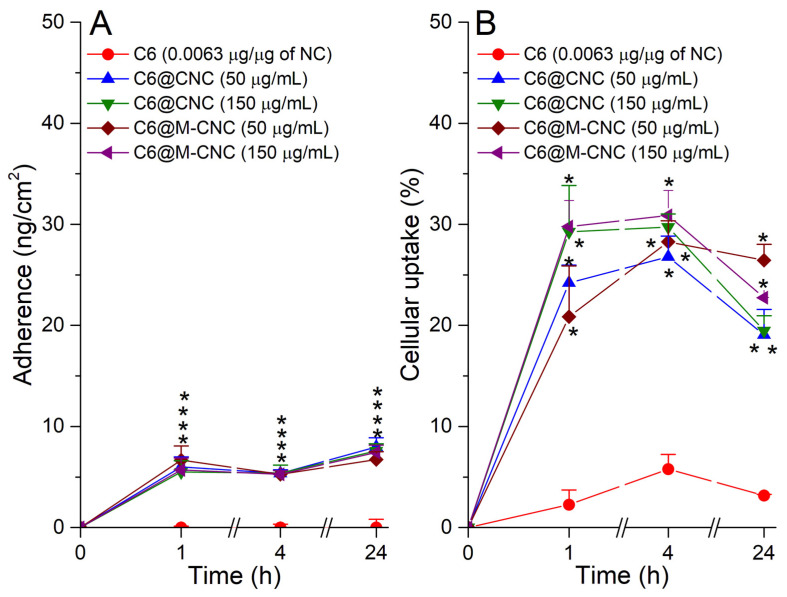
(**A**) Adherence and (**B**) cellular uptake of C6@CNCs and C6@M-CNCs in Caco-2 cells for 50 and 150 µg/mL concentrations. Free C6 (0.0063 µg/µg of NC) was used as a control. Results are expressed as a % of negative control (0 µg/mL) and plotted as mean ± SEM (*n* = 3). Data points significantly different (*p* < 0.05) from the C6-only values are marked with an asterisk.

While a slight increase in the adherence of C6@M-CNCs to Caco-2 cells compared to C6@CNCs was observed, overall results were found to be similar up to 4 h with a slight increase in adherence at the lower concentration (50 µg/mL) for both types of CNCs (Figure 7A). However, the influence of mannosylation was apparent at 24 h where adherence was more pronounced for C6@CNCs compared to C6@M-CNCs at both concentrations (50 and 150 µg/mL). Such observations corroborate well with previous findings [35]. The lower adherence for C6@M-CNCs further confirmed the reduction of free amine groups and, hence, decreased cationic charge due to mannosylation [36].

Caco-2 cells incubated with free C6 showed minimal cellular uptake at all time points (*t* = 1, 4, and 24 h), which indicated that its cellular uptake would only be possible with the assistance of NCs. Interestingly, C6@CNCs displayed a 20–30% cellular uptake while the C6@M-CNCs demonstrated a higher uptake at 24 h than C6@CNCs at 50 µg/mL (Figure 7B). However, there was no significant difference between the C6@CNC and C6@M-CNC at 150 µg/mL after 1, 4, and 24 h. An uptake of C6@CNCs is often due to transcytosis, as reported earlier [37], where the CNCs were endocytosed with a size threshold of 150 nm.

### 3.7. TEER Studies on Caco-2 Monolayers

**Figure 8:**
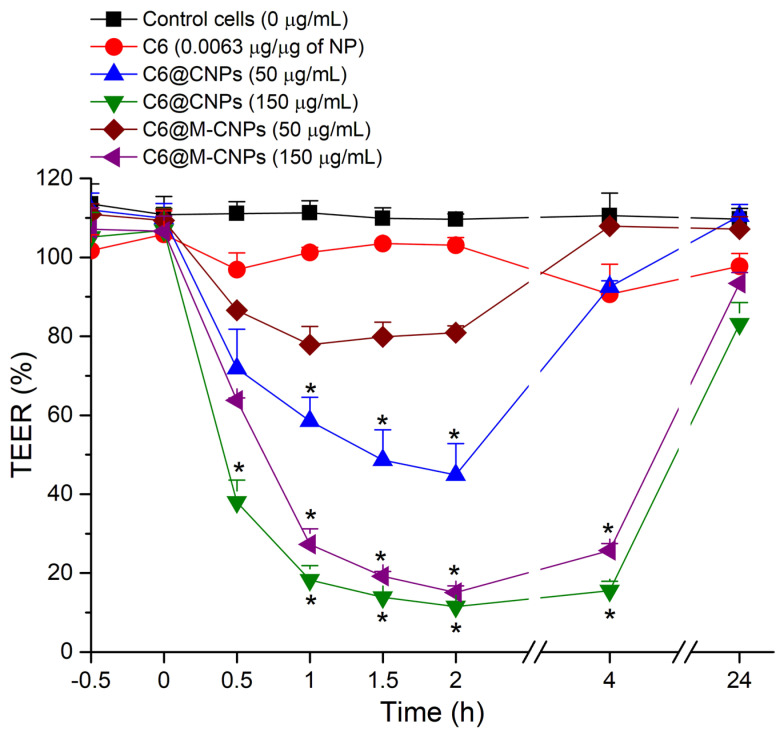
TEER measurements of the Caco-2 monolayers exposed to C6@CNCs and C6@M-CNCs (50 and 150 µg/mL). Results were expressed as % of negative control (0 µg/mL) and are shown as mean ± SEM (*n* = 3). Data points significantly different (*p* < 0.05) from negative control (0 µg/mL) are marked with an asterisk.

The tight junctions in the Caco-2 monolayer have both protective and functional roles. Thus, TEER measurement was used to assess the active involvement of cellular tight junctions in the translocation of NCs while also providing information on membrane integrity (Supplementary Material S5) [38]. A basal TEER of 2605 ± 48 Ω.cm^2^ at 21 days indicated differentiated Caco-2 monolayers on filters [39]. The TEER values of control monolayers exposed to HBSS remained unaffected over 4 h. When the monolayers were exposed to free C6, there was no substantial drop in TEER (Figure 8), whereas, upon exposure to C6@CNCs (50 µg/mL and 150 µg/mL), a 10–15% drop in TEER occurred after 30 min, which was more obvious for the higher concentration (150 µg/mL > 50 µg/mL). However, no further drop in TEER was noted at 4 h. It is known that, like other cationic polymeric NCs [11], CNCs also modulate the tight junctions of a Caco-2 monolayer in a reversible manner [40,41]. A similar trend was observed for C6@M-CNCs over 2 h. However, there was a significant difference in TEER values in the case of C6@M-CNCs compared to the C6@CNCs. In fresh media, recovery of the TEER values was seen at 24 h and it reverted to that of the baseline (negative control). In fact, most of the time point measurements recovered even at 4 h, except for the 150 µg/mL concentration.

### 3.8. Transport of the C6@CNCs and C6@M-CNCs across the Caco-2 Monolayers

**Figure 9:**
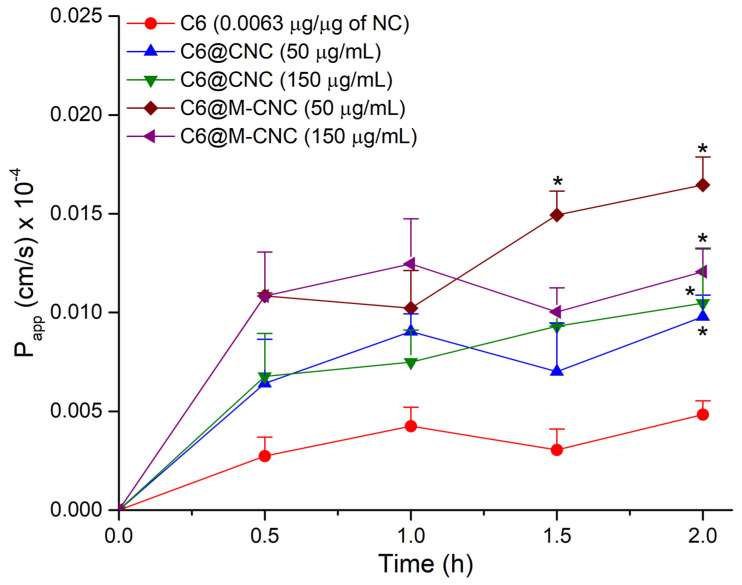
The P_app_ of C6 from C6@CNCs and C6@M-CNCs (50 and 150 µg/mL) across Caco-2 monolayers. Results are presented as mean ± SEM (*n* = 3). Data points significantly different (*p* < 0.05) from the corresponding C6-only values are marked with an asterisk.

Both the C6@CNCs and C6@M-CNCs demonstrated a low flux across monolayers (Figure 9). It is worth mentioning here that the C6 was encapsulated within cross-linked stable NCs with <1% release after 4 h. Hence, the obtained signal reflected the C6 encapsulated within the NCs and not free C6. The P_app_ for C6@CNCs at *t* = 2 h was 9.8 × 10^−7^ cm/s (50 µg/mL) and 1.0 × 10^−6^ (150 µg/mL). On the contrary, the C6@M-CNCs elicited a higher P_app_ of 1.6 × 10^−6^ cm/s (50 µg/mL) and 1.2 × 10^−6^ cm/s (150 µg/mL) at 2 h, suggesting transcellular permeation. Available literature has established the permeation-enhancing role of chitosan and its derivatives—both as a free polymer and NCs [42]. Despite an unclear cellular uptake and transport mechanism, both the C6@CNCs and C6@M-CNCs did not disrupt the Caco-2 monolayer integrity. Previous reports on similar Caco-2 monolayers have claimed that molecules with a P_app_ ≥ 14.0 × 10^−6^ cm/s are regarded as being highly permeable whereas a P_app_ of < 5.0 × 10^−6^ cm/s depicts low permeability. Thus, both prototypes under investigation demonstrated low permeability. Increased surface hydrophilicity is known to facilitate the transport of NCs across Caco-2 monolayers [43]. Thus, the mannosylation-induced increase in hydrophilicity can explain the higher P_app_ for C6@M-CNCs.

### 3.9. Mucoadhesion Studies on Porcine Gut Mucin

**Figure 10:**
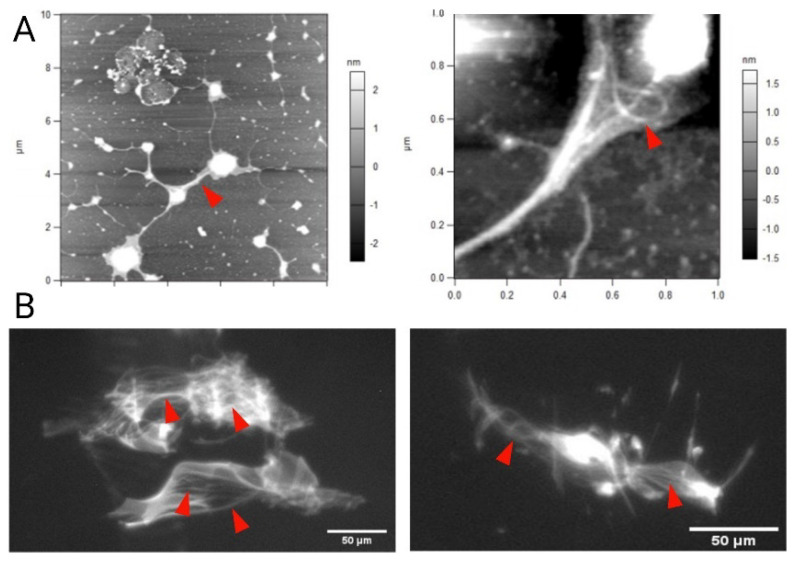
(**A**) The AFM study with height trace of mucin fibers deposited on a mica surface showing the mucin fibers (marked with red arrowhead), individually and in agglomeration with others, thus forming bundles, viewed on a larger field (**left** panel) and a closer zoomed-in view (**right** panel). The scale bar is provided at the sides of the images. (**B**) The epifluorescence images of (both **left** and **right** panels) mucin fibers bound to C6@CNCs. The red arrowheads mark the mucin fibers. No such binding was observed for the C6@M-CNCs. Scale bars (50 µm) are embedded within the images. The reference AFM data helped to exclude any agglomeration of C6@CNCs providing noise.

Due to a smaller size, it is easier for the NCs to permeate through a mucin mesh than microparticles. While providing a structural template to the viscoelastic attributes of mucus hydrogel, the mucin mesh is dynamic, and the fenestrations between adjacent mucin fibers vary between 100 and 200 nm [44]. Atomic force microscopy (AFM) studies confirmed the presence of nanoscale mucin fibers with occasional bundling up of the fibers in the mucin samples (Figure 10A). When mixed with a mucin suspension (1 mg/mL), the C6@CNCs (150 µg/mL) showed instantaneous binding with the mucin fibers. Imagery acquisition of the binding could be achieved (Figure 10B) with epifluorescence microscopy (*λ_ex/em_* = 470/525 nm). On the contrary, the C6@M-CNCs exhibited lesser binding with the mucin, and particle tracking experiments affirmed the flow of these C6@M-CNCs in (mostly) unbound state (Supplementary Material S6).

The disparate mucoadhesion noted for C6@CNCs and C6@M-CNCs can be attributed to a varied electrostatic interaction between the mucin mesh and NCs [44]. It is known that mucin is overall anionic at neutral pH, whereas the C6@CNCs were cationic in aqueous dispersions due to protonation of the terminal amine groups (–NH_3_^+^) in chitosan [45]. Thus, the C6@CNCs showed higher binding to mucin. On the contrary, the net cationic charge on C6@M-CNCs was (partially) masked due to mannosylation, and the terminal hydroxyl groups of mannose ligands imparted anionic charge. Moreover, bioconjugation to a hydrophilic molecule such as mannose augmented the hydrophilicity of native chitosan polymer and, thus, dragged a meniscus of water along with the particles while permeating through mucin—a phenomenon known as the *hydrophilic brush effect* [46,47]. The driving mechanism behind such an effect is still poorly understood despite the effect being noted clearly in viral particles (e.g., poliovirus, Norwalk virus, and poxvirus) that cross the mucus barrier in human bodies almost as efficiently as free diffusion in water [48]. It is hypothesized that high surface charge density on the viral surfaces facilitates its permeation through the mucus by reduced binding. The same mechanism can also explain the lesser mucoadhesion observed for the C6@M-CNCs.

## 4. Conclusions

Fluorescent crosslinked chitosan and mannosylated chitosan NCs with an encapsulated fluorophore (C6) were prepared. The stability of these NCs in conjunction with their adequate biocompatibility and capacity to entrap a hydrophobic macromolecule make them potentially exciting agents for (oral) drug delivery. The flux across Caco-2 monolayers for C6 from both these NC prototypes was evident, with the mannosylated prototype having an increased P_app_ over the non-mannosylated chitosan. Moreover, the C6@M-CNCs exhibited negligible mucoadhesion due to increased hydrophilicity, whereas the CNCs elicited rapid binding to mucin. The C6@M-CNC emerged as a more suitable nano-DDS compared to non-mannosylated C6@CNC. The integrated data highlight the challenges in achieving a delicate balance between stability and optimized release kinetics of CNCs with an entrapped payload. Further optimization of the synthesis and encapsulation of theranostic molecules within modified CNCs are currently being investigated in our labs.

## Data Availability

All the data were reported in the manuscript and further information may be obtained from S.B. upon reasonable request.

## Supplementary Materials

The following are available online at https://www.mdpi.com/article/10.3390/pharmaceutics14040830/s1, Figure S1: Calibration curve for quantification of C6 based on emission spectrum; Figure S2: The principle of transwell experiments with Caco-2 monolayers; Figure S3: Stacked ^1^H spectra of chitosan, mannose, and mannosylated chitosan; Figure S4: Entrapment efficiency of C6 in C6@CNCs and C6@M-CNCs; Figure S5: Brightfield and epifluorescence microscopy on Caco-2 monolayers; Video S6: Single particle tracking of C6@M-CNCs in mucin.
