# Supplementary material for: Add Sugar to Chitosan: Mucoadhesion and In Vitro Intestinal Permeability of Mannosylated Chitosan Nanocarriers
Source: Pharmaceutics. 2022 Apr 11;14(4):830. doi: 10.3390/pharmaceutics14040830 (PMC9024478; doi:10.3390/pharmaceutics14040830)
Supplement: Supplementary file 1 [file pharmaceutics-14-00830-s001.zip › Supplementary Materials.pdf]

SUPPLEMENTARY MATERIALS

Add sugar to chitosan: mucoadhesion and *in vitro* intestinal permeability of mannosylated chitosan nanocarriers

Sadaf Ejaz,[1] Bridget Hogg,[2] Delyan R. Hristov,[2] David J. Brayden,[2,3] Muhammad Imran,[1,*] Sourav Bhattacharjee[2,*]

[1]Department of Biosciences, COMSATS University Islamabad (CUI), Park Road, Islamabad, Pakistan

[2]School of Veterinary Medicine, University College Dublin (UCD), Belfield, Dublin 4, Ireland

[3]Conway Institute of Biomolecular and Biomedical Research, University College Dublin (UCD), Belfield, Dublin 4, Ireland

[*]Corresponding authors:

Muhammad Imran (Email: m.imran@comsats.edu.pk; T.: +92 322 6017784)

Sourav Bhattacharjee (Email: sourav.bhattacharjee@ucd.ie; T.: +353 1 716 6271)

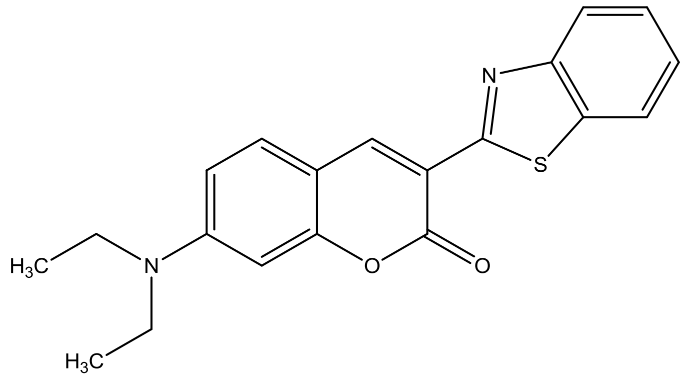

Molecular structure of C6

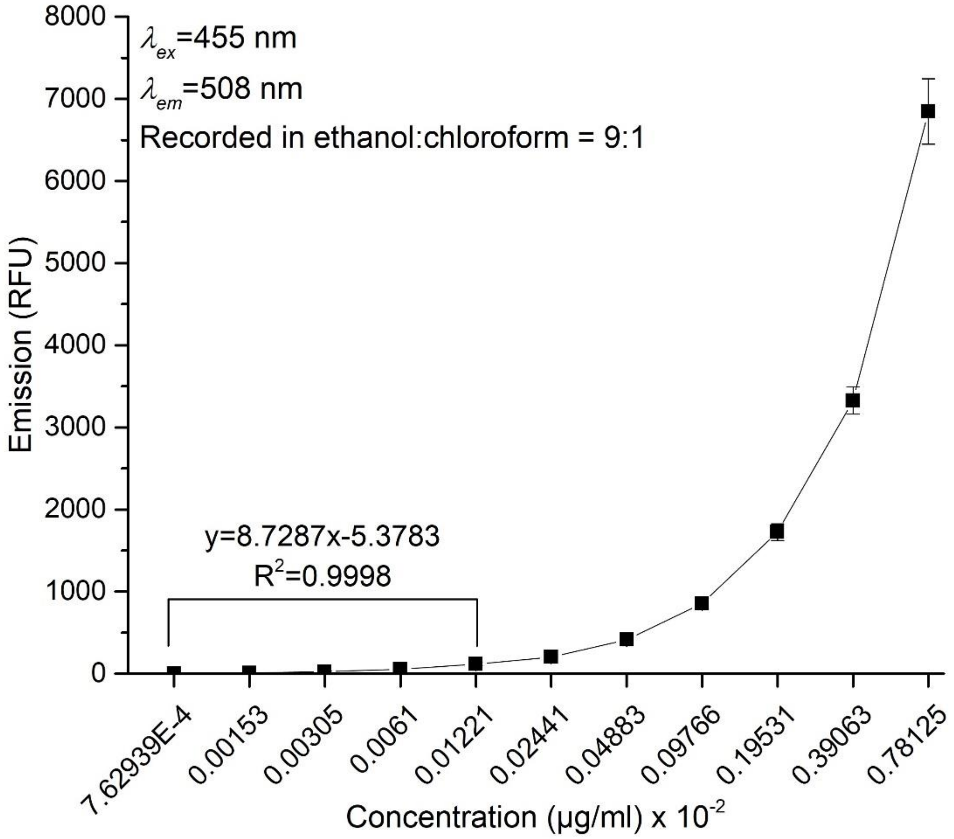

Figure S1: Development of a calibration curve for quantification of C6 based on emission spectrum. Results are shown as mean ± SD (n=2), and the equation of calibration curve is given within the figure.

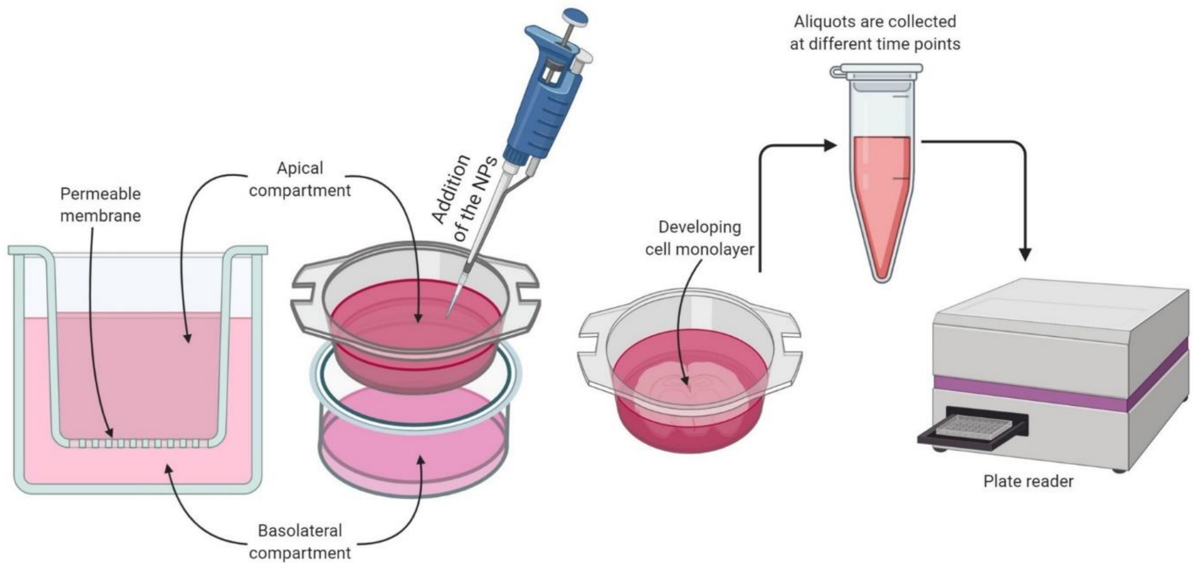

Figure S2: The principle of transwell experiments with Caco-2 monolayers.

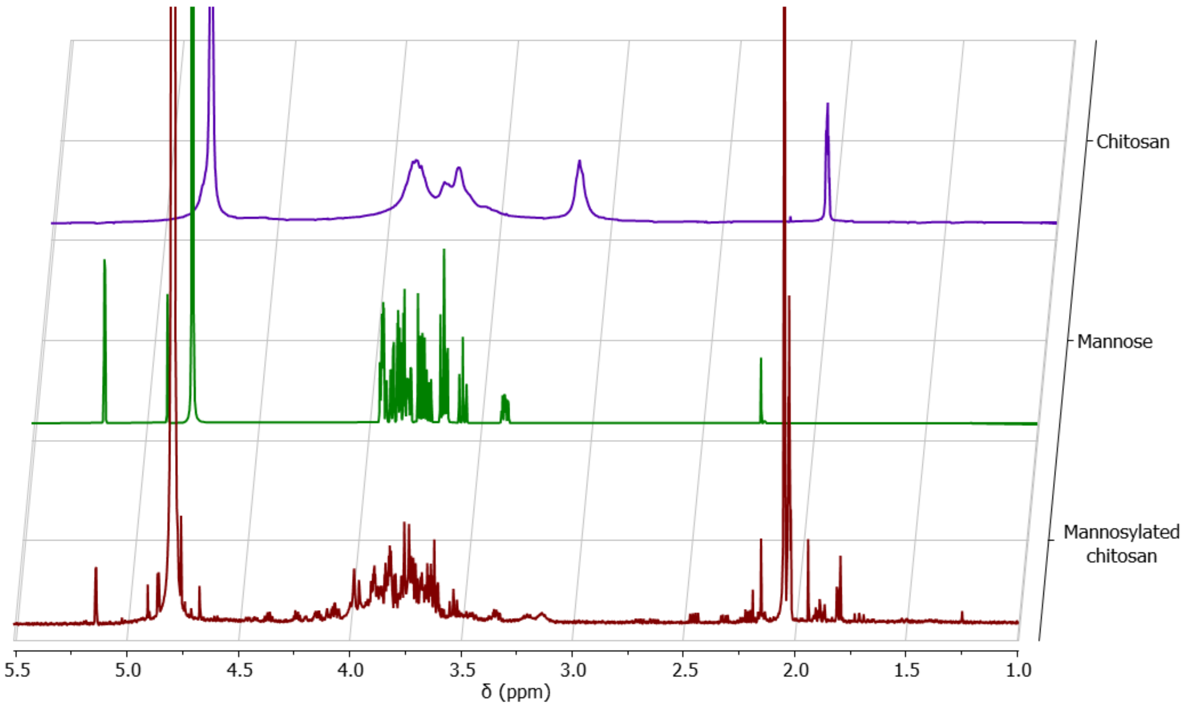

Figure S3: Stacked ^{1}H spectra of chitosan, mannose and mannosylated chitosan.

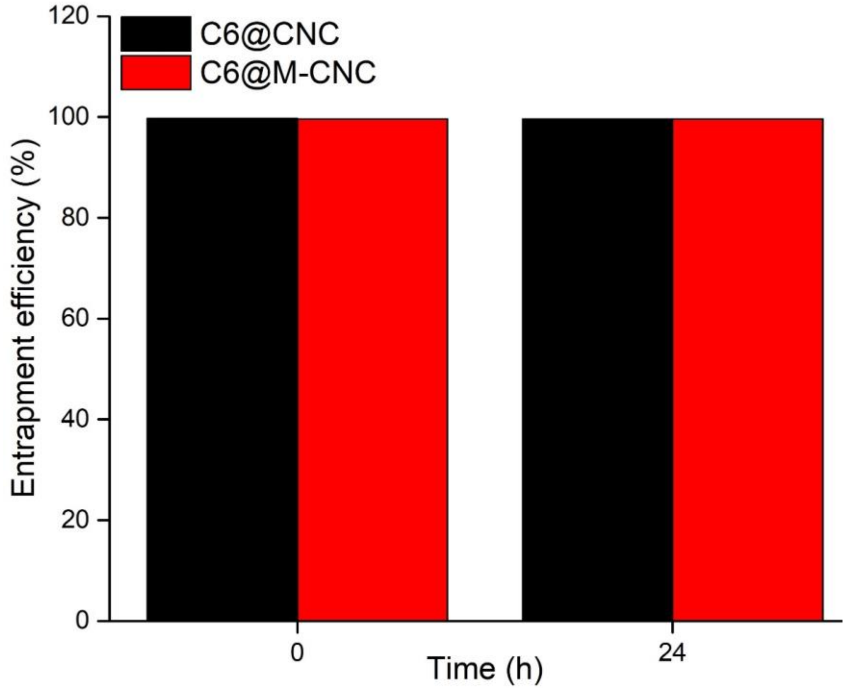

Figure S4: Entrapment efficiency (%) of C6 in C6@CNCs and C6@M-CNCs. Results are shown as mean ± SD (n=3).

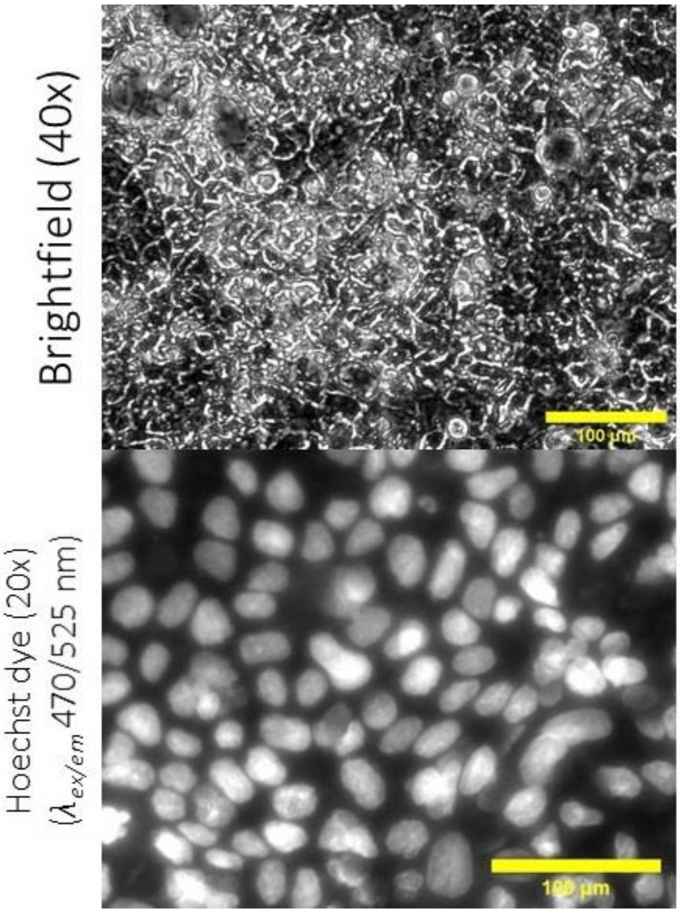

Figure S5: The brightfield (upper layer, 40×) and epifluorescence microscopy ($\lambda_{ex/em}$=470/525 nm, 20×, lower layer) on Caco-2 monolayers stained with Hoechst dye to image the cellular nuclei. Scale bars (100 µm) are embedded within the figures.
